# Variability in exercise tolerance and physiological responses to exercise prescribed relative to physiological thresholds and to maximum oxygen uptake

**DOI:** 10.1113/EP090878

**Published:** 2023-01-29

**Authors:** Samuel Meyler, Lindsay Bottoms, David Wellsted, Daniel Muniz‐Pumares

**Affiliations:** ^1^ School of Life and Medical Sciences University of Hertfordshire Hatfield UK

**Keywords:** critical power, exercise intensity, exercise prescription, interindividual differences

## Abstract

The objective of this study was to determine whether the variability in exercise tolerance and physiological responses is lower when exercise is prescribed relative to physiological thresholds (THR) compared to traditional intensity anchors (TRAD). Ten individuals completed a series of maximal exercise tests and a series of moderate (MOD), heavy (HVY) and severe intensity (HIIT) exercise bouts prescribed using THR intensity anchors (critical power and gas exchange threshold) and TRAD intensity anchors (maximum oxygen uptake; ${\dot{V}}_{O_{2}\max}$). There were no differences in exercise tolerance or acute response variability between MOD_THR_ and MOD_TRAD_. All individuals completed HVY_THR_ but only 30% completed HVY_TRAD_. Compared to HVY_THR_, where work rates were all below critical power, work rates in HVY_TRAD_ exceeded critical power in 70% of individuals. There was, however, no difference in acute response variability between HVY_THR_ and HVY_TRAD_. All individuals completed HIIT_THR_ but only 20% completed HIIT_TRAD_. The variability in peak (*F* = 0.274) and average (*F* = 0.318) blood lactate responses was lower in HIIT_THR_ compared to HIIT_TRAD_. The variability in *W′* depletion (the finite work capacity above critical power) after the final interval bout was lower in HIIT_THR_ compared to HIIT_TRAD_ (*F* = 0.305). Using physiological thresholds to prescribe exercise intensity reduced the heterogeneity in exercise tolerance and physiological responses to exercise spanning the boundary between the heavy and severe intensity domains. To increase the precision of exercise intensity prescription, it is recommended that, where possible, physiological thresholds are used in place of ${\dot{V}}_{O_{2}\max}$.

## Abstract (highlights)

- **What is the central question of this study?**
- Does prescribing exercise intensity using physiological thresholds create a more homogeneous exercise stimulus than using traditional intensity anchors?
- **What is the main finding and its importance?**
- Prescribing exercise using physiological thresholds, notably critical power, reduced the variability in exercise tolerance and acute metabolic responses. At higher intensities, approaching or exceeding the transition from heavy to severe intensity exercise, the imprecision of using fixed %${\dot{V}}_{O_{2}\max}$ as an intensity anchor becomes amplified.

## 1 INTRODUCTION

Cardiorespiratory fitness, measured as maximum oxygen uptake (${\dot{V}}_{O_{2}\max}$), is an important marker of both endurance performance (Bassett & Howley, 2000) and cardiovascular health (Harber et al., 2017). The most effective means of increasing ${\dot{V}}_{O_{2}\max}$ is via endurance training (ET), encompassing high intensity interval training and/or continuous exercise (Milanović et al., 2015). However, the effect of ET on ${\dot{V}}_{O_{2}\max}$ appears to be largely heterogeneous among individuals (Bouchard et al., 1999; Williams et al., 2019).

Many factors may contribute to ${\dot{V}}_{O_{2}\max}$ response variability. Some relate to unmodifiable factors, such as age and genetics, and some to modifiable factors, such as training characteristics, whilst others relate to measurement error and biological variability (Bonafiglia et al., 2022; Meyler et al., 2021). A modifiable factor of interest is *how* exercise intensity is prescribed, which, when manipulated, may reduce response variability by creating a more homogeneous exercise and training stimulus among individuals (Meyler et al., 2021). Improving exercise intensity prescription reflects a subtractive approach that may be a means of reducing response variability without having to exhaust additive approaches where additional stimuli are needed (Adams et al., 2021), for example, by increasing training dose (Bonafiglia et al., 2021).

Exercise intensity is prescribed along a continuum of intensity domains partitioned into moderate, heavy (vigorous) and severe (near‐maximal to maximal), each of which is associated with domain‐specific metabolic and cardiopulmonary responses (Black et al., 2017; Carter et al., 2002). Notably, these domains are delineated by physiological thresholds, whereby the transition between the moderate and heavy domain and the heavy and severe domain can be determined by the gas exchange threshold (GET) and critical power (CP), respectively (Poole et al., 2020; Wasserman et al., 1973). To target each intensity domain and the associated exercise stimuli, intensity is commonly prescribed as a fixed %${\dot{V}}_{O_{2}\max}$ (Milanović et al., 2015; Williams et al., 2019). However, among individuals, this approach elicits marked variations in the acute physiological responses to exercise and time to task failure despite undertaking exercise at the ‘same’ relative intensity (Baldwin et al., 2000; Iannetta et al., 2020; Lansley et al., 2011; Meyer et al., 1999; Scharhag‐Rosenberger et al., 2010).

Alternatively, using physiological thresholds to prescribe exercise may improve intensity normalisation among individuals as they consider the size and positioning of an individual's intensity domains relative to ${\dot{V}}_{O_{2}\max}$. Compared to exercise prescribed relative to ${\dot{V}}_{O_{2}\max}$, more homogeneous physiological responses have been observed when exercise is prescribed relative to physiological thresholds such as GET (Lansley et al., 2011), lactate threshold (Baldwin et al., 2000) and the onset of blood lactate accumulation (McLellan & Jacobs, 1991). As it has recently been argued that CP is the most accurate delineator of the transition between the heavy and severe intensity domains (Jones et al., 2019), using CP as an anchor of exercise intensity might improve intensity normalisation among individuals when exercising at higher intensities (Collins et al., 2022). However, exploring the variability in exercise tolerance and acute physiological responses to exercise prescribed relative to CP compared to traditional intensity anchors is yet to be investigated. Nor has the magnitude of variability been explored in relation to interval‐based exercise. Additionally, it is of interest to determine the variability in how exhaustive interval bouts are among individuals. This can be achieved by modelling the depletion of an individual's finite work capacity (*W′*) that exists at intensities exceeding critical power (Skiba & Clarke, 2021).

The purpose of this study was, therefore, to compare the variability in acute physiological responses to moderate intensity continuous exercise, heavy intensity continuous exercise and high intensity interval exercise prescribed relative to ${\dot{V}}_{O_{2}\max}$ (TRAD), and to GET and CP (THR). We hypothesised that the magnitude of variability in the acute physiological responses to exercise would be lower among individuals when exercise is prescribed using THR compared to TRAD approaches.

## 2 METHODS

### 2.1 Ethical approval

The study was approved by the University of Hertfordshire Health, Science, Engineering and Technology Ethics Committee and Delegated Authority (protocol: LMS/PGR/UH/04708) and was conducted in accordance with the *Declaration of Helsinki*, except for registration in database. All participants provided written informed consent.

### 2.2 Participants

Ten healthy, recreationally active individuals volunteered to participate in the study (Table 1). Participants were 18+ years old, non‐smokers, non‐obese (BMI < 30 kg m^−2^), and free from any disease and musculoskeletal injuries.

**TABLE 1:** Participant characteristics.

Sex	Males (*n* = 7)	Females (*n* = 3)	Total (*n* = 10)
Age (years)	22 ± 4	26 ± 9	23 ± 6
Height (cm)	180 ± 8	168 ± 5	176 ± 9
Mass (kg)	84 ± 13	63 ± 8	78 ± 15
BMI (kg m^−2^)	26 ± 4	22 ± 3	25 ± 4
${\dot{V}}_{O_{2}\max}$ (ml kg^−1^ min^−1^)	37 ± 5	40 ± 3	38 ± 4
${\dot{V}}_{O_{2}\max}$ (l min^−1^)	3.11 ± 0.35	2.52 ± 0.12	2.93 ± 0.41

Data are reported means ± SD. Abbreviations: BMI, body mass index; ${\dot{V}}_{O_{2}\max}$, maximum oxygen uptake.

### 2.3 Experimental design

**FIGURE 1:**
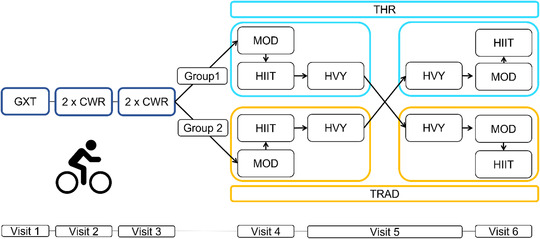
Experimental study protocol. CWR, constant work rate tests; GXT, graded maximal ramp exercise test; HIIT, high‐intensity interval training; HVY, heavy intensity continuous exercise; MOD, moderate intensity continuous exercise; THR, threshold‐based exercise; TRAD, traditionally prescribed exercise.

This study implemented a randomised cross‐over design. Participants visited the laboratory six times (Figure 1) undergoing a block of exercise testing (visits 1–3) followed by two batteries of exercise bouts where the intensity was prescribed using both TRAD and THR approaches (visits 4–6). Participants were randomly allocated into two groups. Group 1 performed THR exercise first, followed by TRAD exercise. Group 2 performed TRAD exercise first, followed by THR exercise. Participants were blinded to the experimental conditions being undertaken. Participants were asked to arrive at the laboratory fully rested, and all sessions were performed at similar times of day and separated by a minimum of 24 h. All exercise tests and exercise bouts were performed on an electromagnetically braked cycle ergometer (Excalibur Sport V2, Lode, Groningen, Netherlands).

### 2.4 Exercise testing

#### 2.4.1 Maximal ramp exercise test

On visit one, participants performed a graded maximal ramp exercise test (GXT) to determine GET, ${\dot{V}}_{O_{2}\max}$ and maximum heart rate (HR_max_). Participants completed a standardised warm‐up consisting of 3 min unloaded cycling at a self‐selected cadence (70–90 rpm). Starting at 0 W, work rate increased by 30 W every minute until task failure. Task failure was defined as a decrease in cadence >10 rpm below self‐selected test cadence for >5 s. Breath‐by‐breath pulmonary gas exchange and heart rate (HR) data were collected continuously throughout the test and averaged over 10 s periods. ${\dot{V}}_{O_{2}\max}$ was recorded as the highest mean ${\dot{V}}_{O_{2}}$ during a 30 s period and GET as the first disproportionate increase in carbon dioxide production (${\dot{V}}_{CO_{2}}$) from visual inspection of individual ${\dot{V}}_{CO_{2}}$ versus ${\dot{V}}_{O_{2}}$ plots (Keir et al., 2022). GET was then confirmed by visual inspection of additional breath‐by‐breath plots using an online exercise thresholds tool (Keir et al., 2022), and agreement with another researcher (D.M.) was then sought. To verify the attainment of ${\dot{V}}_{O_{2}\max}$, a verification bout (VER), intended to last between 3 and 6 min, was performed following 20 min recovery post‐GXT (Nolan et al., 2014). Work rate was set at 85% of the maximum power output achieved in the GXT and was performed to task failure (Poole & Jones, 2017). The attainment of ${\dot{V}}_{O_{2}\max}$ was assumed if the difference between GXT and VER ${\dot{V}}_{O_{2}\max}$ was ≤5% and the average value of the two tests was taken forward as ${\dot{V}}_{O_{2}\max}$.

#### 2.4.2 Constant work rate tests

On visits two and three, participants performed two constant work rate tests (CWR) per day with an inter‐trial recovery time of 1 h in order to estimate CP and *W′* (Hunter et al., 2021). Each CWR was intended to elicit task failure between 2 and 15 min. Participants completed a 3‐min warm up, cycling at a low work rate of 25 W and self‐selected cadence (70–90 rpm). Work rate was then suddenly increased to the target work rate and participants cycled to task failure at their self‐selected cadence. Attainment of ${\dot{V}}_{O_{2}\max}$ during CWR was again confirmed if ${\dot{V}}_{O_{2}\max}$ was ≤5% of determined ${\dot{V}}_{O_{2}\max}$. To estimate CP and *W′*, three models were used per participant (Muniz‐Pumares et al., 2019) as follows.

1. A non‐linear power‐time model:


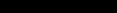

where *T*_lim_ is time to task failure (s), *P* is power output (W), CP is the asymptote of the hyperbolic relationship, and *W′* is the curvature constant.

2. A linear work‐time model:
$$W\;=\;W^{\prime }+\;\mathrm{CP}\;\times \;T_{\lim}$$

using linear regression analysis where *W* is work (kJ), the *y*‐intercept represents *W′*, and the slope represents CP.

3. A linear 1/time model:
$$P\;=\;\mathrm{CP}\;+\;W^{\prime }\times \;{T_{\lim}}^{-1}$$

where the *y*‐intercept represents CP and the slope represents *W′*.

For each participant, the standard error of estimate (SEE) was determined for CP and *W′* and the model producing the lowest combined SEE for each individual was used to estimate CP and *W′* on an individual basis (Black et al., 2017).

### 2.5 Exercise bouts

Intra‐visit exercise bouts were all separated by a 1‐h recovery period. The intensity for exercise bouts was chosen to correspond to moderate (MOD), heavy (HVY) and severe intensity exercise (which was in the form of high intensity interval training; HIIT) (Table 2). MOD_TRAD_ and HVY_TRAD_ were prescribed as the midpoint between the ranges of ${\dot{V}}_{O_{2}\max}$ intended to elicit moderate (46–63%) and heavy (64–90%) intensity exercise, respectively (American College of Sports Medicine, 2017). The HIIT protocols implemented a 1:1 work:rest ratio, with active recovery at 20 W. HIIT exercise bouts were designed based on the findings of Wen et al. (2019) whereby long intervals (≥2 min) and high volumes (≥15 min) at 80–90% ${\dot{V}}_{O_{2}\max}$ are recommended to maximise training effects on ${\dot{V}}_{O_{2}\max}$. The power output for both HIIT_THR_ and HIIT_TRAD_ was intended to correspond to severe intensity exercise. When following the American College of Sports Medicine (ACSM) guidelines on severe intensity exercise, intensities of ≥91% ${\dot{V}}_{O_{2}\max}$ are proposed. However, following pilot testing this was not suitable when trying to complete ≥2 min intervals. Therefore, the intensity for HIIT_TRAD_ was reduced to 85% ${\dot{V}}_{O_{2}\max}$ (‘heavy’ intensity exercise according to the ACSM guidelines; American College of Sports Medicine, 2017). The work rate in TRAD sessions was extrapolated from the ${\dot{V}}_{O_{2}}$–intensity relationship derived from the GXT, with the first minute of test ${\dot{V}}_{O_{2}}$ data being removed from the calculation (Keir et al., 2022).

**TABLE 2:** Prescribed exercise bouts.

	THR	TRAD
MOD	30 min @ 90% GET	30 min @ 55% ${\dot{V}}_{O_{2}\max}$
HVY	20 min @ 50% ∆ (GET + [0.5 × (CP – GET)])	20 min @ 75% ${\dot{V}}_{O_{2}\max}$
HIIT	5 × 3 min @ 110% CP	5 × 3 min @ 85% ${\dot{V}}_{O_{2}\max}$

50% ∆ is power at GET + 50% difference between GET and CP. Abbreviations: CP, critical power; GET, gas exchange threshold; HIIT, high‐intensity interval training; HVY, heavy intensity continuous exercise; MOD, moderate intensity continuous exercise; THR, exercise prescribed relative to physiological thresholds; TRAD, exercise prescribed relative to ${\dot{V}}_{O_{2}\max}$; ${\dot{V}}_{O_{2}\max}$, maximum oxygen uptake.

### 2.6 Utilisation of the *W′* balance model

The *W′*_BAL‐INT_ model (Skiba & Clarke, 2021) was used to determine how much of the work capacity above CP (*W′*) was depleted during the HIIT exercise bouts. *W′*_BAL‐INT_ was calculated to the end of the final HIIT bout or at task failure, whichever was sooner. *W′*_BAL‐INT_ was calculated as:

$$W_{\mathrm{BAL}-\mathrm{INT}}^{\prime }\;\left(t\right)=\;W_{0}^{\prime }-\;\int_{0}^{t}\left[e^{\left(-\frac{t-u}{\tau_{W^{\prime }}}\right)}\right]W_{\mathrm{EXP}}^{\prime }\;\left(u\right)\;du$$

where *W′*_BAL‐INT_ (*t*) is the amount of *W* *′* remaining at any given time *t*, *W′* is the individual's known *W′*. *W′*_EXP_ represents the expended *W′*, *t* and *u* represent time, and $\tau_{W}$ is the time constant of the reconstitution of the *W′*. *W′*_EXP_ (u) is calculated as:

$$W_{\mathrm{EXP}}^{\prime }\;\left(u\right)=\;\left\{\begin{array}{c} 0,\;P\left(u\right)\;\leq \mathrm{CP}\\ \int \left(P\left(u\right)-\mathrm{CP}\right)du,\;P\left(u\right)>\mathrm{CP} \end{array}\right.$$

and:

$$\;\tau_{W^{\prime }}=\;546\;\times \;e^{\left(-0.01D_{\mathrm{CP}}\right)}+316$$

where *D*_CP_ is the difference between CP and the power output (*P*) during the recovery period.

### 2.7 Measurements

During all exercise tests and exercise bouts, gas exchange data were measured continuously breath‐by‐breath using an online gas analyser (MetaLyzer 3B, Cortex Biophysik, Leipzig, Germany). Participants wore a face mask with low dead space (125 ml) and breathed through a low resistance (<0.1 kPa l^−1^ at 20 l s^−1^) impeller turbine with O_2_ and CO_2_ samples at 50 Hz. The gas analyser was calibrated prior to each exercise session with gases of known concentration, and the turbine volume transducer was calibrated using a 3‐litre syringe (Hans Rudolph, Inc., Kansas City, MO, USA). Rise time of the gas analyser and transit delay for O_2_ and CO_2_ were <100 ms and 800–1200 ms, respectively, allowing for breath‐by‐breath calculation. Measurements of ${\dot{V}}_{O_{2}}$ and ${\dot{V}}_{CO_{2}}$ were recorded breath‐by‐breath and exported as 10‐s moving averages for subsequent analyses. Heart rate was measured telemetrically throughout the exercise session and exported as 10‐s moving averages for subsequent analyses (Polar H10, Polar Electro, Kempele, Switzerland). During the exercise bouts, capillary blood samples (10 μl) were taken from the fingertip and analysed (Biosen C‐Line, EKF Diagnostics, Cardiff, UK) to determine blood lactate concentration (BLa). For MOD and HVY, blood samples were taken at rest, during the last 30 s of the warm‐up, and then every 5 min for the remainder of the exercise bout or at task failure. During HIIT, blood samples were taken at rest and at the start of each recovery period or until task failure.

### 2.8 Statistical analyses

To evaluate the magnitude of acute physiological response variability, the standard deviation (SD) and mean responses were first calculated for THR and TRAD during MOD, HVY and HIIT exercise bouts. The SD values were then compared between THR and TRAD sessions using the *F‐*distribution. Where data for an individual were missing (i.e., at time points after a premature cessation of exercise) a sensitivity analysis was conducted to determine the effect of different assumptions about the missing values on the mean to avoid missing data biasing conclusions based on observed data. Taking into consideration the sample size of the current study (*n* = 10), interpretation of the comparison between variances will consider both the *P‐*value and the magnitude of the *F* ‐ ratio as an indicator of the magnitude of difference. As the *F‐*test is being used with *n* = 10, the *F‐*statistic will be treated as an effect size estimator, and any ratio <0.33 will be considered of sufficient magnitude to indicate a difference that could potentially be significant with a larger sample (Chen & Chen, 2010). This approach helps protect against accepting the null hypothesis when there is a lack of power to truly evaluate the difference. The chi square test was used to compare the proportion of individuals completing THR and TRAD sessions. Differences in group means were compared using Student's *t‐*test. Significance was accepted when *P* < 0.05. Statistical analyses were conducted using R (version 4.2.0; R Foundation for Statistical Computing, Vienna, Austria) and JASP (version 0.16.2).

## 3 RESULTS

### 3.1 Exercise tests

In the GXT and the verification test, the highest ${\dot{V}}_{O_{2}}$ recorded over a 30 s period was 38 ± 4 ml kg^−1^ min^−1^ (2.95 ± 0.43 l min^−1^) and 38 ± 4 ml kg^−1^ min^−1^ (2.91 ± 0.39 l min^−1^), respectively, with a difference of 1 ± 3% (range: −2 to 5 ml kg^−1^ min^−1^). Therefore, ${\dot{V}}_{O_{2}\max}$ was calculated as the average of values attained in the GXT and verification test. Peak power output in GXT was 292 ± 33 W. Power output at GET was 113 ± 17 W and occurred at 52 ± 4% ${\dot{V}}_{O_{2}\max}$.

Power output at CP was 172 ± 27 W and occurred at 69 ± 6% ${\dot{V}}_{O_{2}\max}$. GET occurred at 67 ± 12% CP. The highest ${\dot{V}}_{O_{2}}$ attained in all CWR trials was 39 ± 5 ml kg^−1^ min^−1^ (3.02 ± 0.44 l min^−1^) which was not different from ${\dot{V}}_{O_{2}\max}$ (*P* = 0.954). For individuals where linear work‐time CP model was used (*n* = 9), fits were r^2^ = 0.99. The linear 1/Time model was used for the remaining individual (*n* = 1) where the fit was r^2^ = 0.99. Shortest time to exhaustion CWR trials were 196 ± 36 s and longest were 796 ± 167 s.

### 3.2 Exercise bouts

Summary data for each exercise bout are presented in Table 3. Completion rates for MOD_TRAD_ and MOD_THR_ were 100%. Completion rates were lower for HVY_TRAD_ compared to HVY_THR_ (30% vs. 100%, *P* < 0.001) and for HIIT_TRAD_ compared to HIIT_THR_ (20% vs. 100%, *P* < 0.001). The percentage of the HVY_TRAD_ and HIIT_TRAD_ completed ranged between 32% and 100% (387–1200 s) and 17% and 100% (310–1800 s), respectively. There was no difference in work rate variance expressed as a percentage of CP between MOD_THR_ and MOD_TRAD_ (60 ± 11 vs. 73 ± 9; *F* = 1.412); however, the variability was lower in HVY_THR_ compared to HVY_TRAD_ (83 ± 6 vs. 113 ± 13; *F* = 0.234) and in HIIT_THR_ compared to HIIT_TRAD_ (110 ± 0 vs. 134 ± 15; *F* < 0.001). Expressed as a percentage of CP, intensities ranged between 45% and 79% and 57% and 85% in MOD_THR_ and MOD_TRAD_, respectively, 75% and 94% and 96% and 132% in HVY_THR_ and HVY_TRAD_, respectively, and 110 ± 0% and 115% and 156% in HIIT_THR_ and HIIT_TRAD_, respectively.

**TABLE 3:** Summary of group data from exercise bouts.

Exercise bout	Work rate (W)	Work rate (%CP)	*F* ‐ ratio	Individuals completing exercise bout (%)	*P*	Percentage of exercise bout completed
MOD_THR_	102 ± 15	60 ± 11	1.412	100	—	100
MOD_TRAD_	124 ± 14	73 ± 9		100		100
HVY_THR_	143 ± 18	83 ± 6^†^	0.234	100*	<0.001	100
HVY_TRAD_	193 ± 19	113 ± 13		30		32–100
HIIT_THR_	190 ± 30	110 ± 0^†^	<0.001	100*	<0.001	100
HIIT_TRAD_	228 ± 23	134 ± 15		20		17–100

* Significant difference between THR and TRAD (*P* < 0.05).

^†^ Variance is significantly lower in THR group compared to TRAD (*F* < 0.33). *n* = 10. Abbreviations: HIIT, high intensity interval training; HVY, heavy intensity exercise bout; MOD, moderate intensity exercise bout; THR, threshold‐based exercise intensity prescription; TRAD, traditionally prescribed exercise intensity.

Physiological data from all exercise bouts are presented in Table 4. There was no difference in the variability of peak or average ${\dot{V}}_{O_{2}}$, HR or BLa between MOD_THR_ and MOD_TRAD_, or between HVY_THR_ and HVY_TRAD_. There was no difference in the variability of peak or average ${\dot{V}}_{O_{2}}$ or HR between HIIT_THR_ and HIIT_TRAD_. The variability in peak and average BLa was lower in HIIT_THR_ compared to HIIT_TRAD_. *W′* depleted in the first 3‐min interval during the HIIT exercise was greater (*P* < 0.001) in HIIT_TRAD_ (49 ± 7%, 39–58%) compared to HIIT_THR_ (17 ± 7%, 10–30%), and *W′* depleted at the end‐point of exercise was greater (*P* < 0.001) in HIIT_TRAD_ (73 ± 22%, 44–99%) compared to HIIT_THR_ (30 ± 12%, 17–53%). The variability in *W′* depleted at the end of HIIT was lower in HIIT_THR_ compared to HIIT_TRAD_ (*F* = 0.305).

**TABLE 4:** Summary of group physiological data from exercise bouts.

Exercise bout	${\dot{V}}_{O_{2}\mathrm{peak}}$ (l min^−1^)	*F* ‐ ratio	${\dot{V}}_{O_{2}\mathrm{peak}}$ (%${\dot{V}}_{O_{2}\max}$)	*F* ‐ ratio	HR_peak_ (b min^−1^)	*F* ‐ ratio	HR_peak_ (%HR_max_)	*F* ‐ ratio	BLa_peak_ (mmol l^−1^)	*F* ‐ ratio
MOD_THR_	1.77 ± 0.31	0.900	61 ± 9	1.648	140 ± 12	1.085	75 ± 7	1.976	2.95 ± 1.35	0.973
MOD_TRAD_	2.02 ± 0.32		69 ± 7		149 ± 11		80 ± 5		3.82 ± 1.37	
HVY_THR_	2.27 ± 0.37	0.947	78 ± 7	1.777	160 ± 11	0.701	85 ± 5	0.979	4.68 ± 1.48	0.361
HVY_TRAD_	2.80 ± 0.38		96 ± 6		182 ± 13		97 ± 5		9.48 ± 2.46	
HIIT_THR_	2.73 ± 0.37	0.825	93 ± 5	1.116	176 ± 11	1.190	94 ± 6	1.395	7.45 ± 1.70^†^	0.274
HIIT_TRAD_	2.93 ± 0.41		100 ± 5		184 ± 12		98 ± 5		10.91 ± 3.23	

Exercise bout	${\dot{V}}_{O_{2}\mathrm{avg}}$ **(l min^−1^)**	*F* ‐ ratio	${\dot{V}}_{O_{2}\mathrm{avg}}$ **(** **%** ${\dot{V}}_{O_{2}\max}$ **)**	*F* ‐ ratio	HR_avg_ (b min^−1^)	*F* ‐ ratio	HR_avg_ (%HR_max_)	*F* ‐ ratio	BLa_avg_ (mmol l^−1^)	*F* ‐ ratio
MOD_THR_	1.67 ± 0.28	0.708	58 ± 8	1.513	134 ± 13	1.51	71 ± 7	2.351	2.43 ± 1.20	0.874
MOD_TRAD_	1.91 ± 0.33		65 ± 6		143 ± 11		76 ± 5		3.31 ± 1.28	
HVY_THR_	2.20 ± 0.34	0.828	75 ± 6	1.049	154 ± 10	0.657	82 ± 5	1.136	4.12 ± 1.30	0.403
HVY_TRAD_	2.71 ± 0.37		93 ± 6		175 ± 12		94 ± 5		8.06 ± 2.85	
HIIT_THR_	2.61 ± 0.32	0.703	89 ± 5	0.889	171 ± 12	1.031	91 ± 6	1.925	6.50 ± 1.30^†^	0.318
HIIT_TRAD_	2.85 ± 0.38		97 ± 5		179 ± 12		96 ± 6		9.09 ± 2.31	

^†^ Variance is significantly lower in THR group compared to TRAD (*F* < 0.33). *n* = 10. Abbreviations: BLa_avg_, average blood lactate; BLa_peak_, peak blood lactate; HIIT, high intensity interval training; HR_avg_, average heart rate; HR_max_, maximum heart rate; HR_peak_, peak heart rate; HVY, heavy intensity exercise bout; MOD, moderate intensity exercise bout; THR, threshold‐based exercise intensity prescription; TRAD, traditionally prescribed exercise intensity; ${\dot{V}}_{O_{2}\mathrm{avg}}$, average oxygen uptake; ${\dot{V}}_{O_{2}\max}$, maximum oxygen uptake; ${\dot{V}}_{O_{2}\mathrm{peak}}$, peak oxygen uptake.

## 4 DISCUSSION

This study is the first to explore the variability in exercise tolerance and acute physiological responses to moderate, heavy and severe intensity exercise prescribed relative to GET and CP and to ${\dot{V}}_{O_{2}\max}$. When prescribing severe intensity exercise relative to ${\dot{V}}_{O_{2}\max}$, the magnitude of variability in exercise tolerance and metabolic responses was greater than when exercise was prescribed relative to CP. This study demonstrates that using CP to prescribe exercise intensity creates a more homogeneous exercise stimulus among individuals.

**FIGURE 2:**
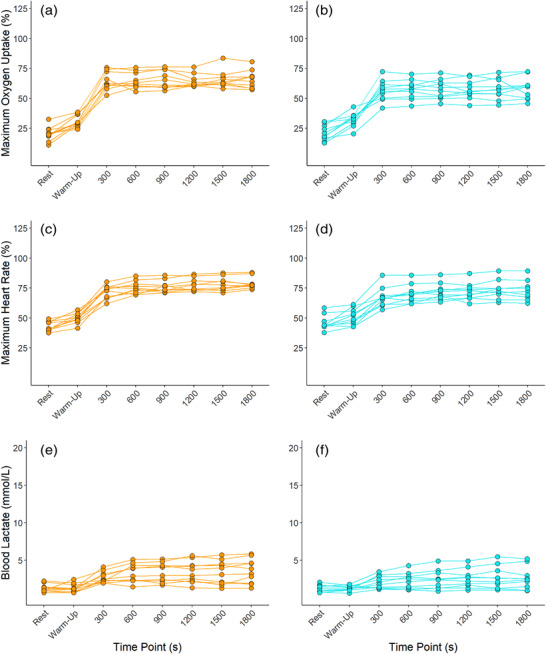
Individual (orange: MOD_TRAD_; blue: MOD_THR_) responses in oxygen uptake expressed relative to maximum oxygen uptake (a, b), heart rate expressed relative to maximum heart rate (c, d), and blood lactate (e, f).

All individuals completed MOD_THR_ and MOD_TRAD_ to their entirety, and the majority displayed physiological response profiles consistent with moderate intensity exercise whereby early physiological steady‐state is attained (Figure 2). Accordingly, in MOD_THR_, only one individual experienced a >1 mmol l^−1^ increase in BLa from 600 s to 1800 s. This supports the findings of McLellan and Jacobs (1991) and Baldwin et al. (2000) who observed no differences in BLa response variability among trained and untrained individuals when exercise was prescribed below the onset of blood lactate accumulation and the lactate threshold, respectively. When exercising at 55% ${\dot{V}}_{O_{2}\max}$ , only four individuals’ work rates were below GET, but the intensity was low enough such that 30 min of exercise could be completed and only one individual experienced an increase in BLa > 1 mmol l^−1^ from 600 s to 1800 s. In the present study, work rates corresponding to 55% ${\dot{V}}_{O_{2}\max}$ and 90% GET were both successful in prescribing continuous exercise that could be tolerated for 30 min. If intensity control is a primary focus, then using GET to prescribe moderate intensity exercise may be more beneficial. Online tools are available to help determine an individual's thresholds from GXT values and should facilitate a switch from using fixed %${\dot{V}}_{O_{2}\max}$ to inform exercise prescription (Keir et al., 2022).

**FIGURE 3:**
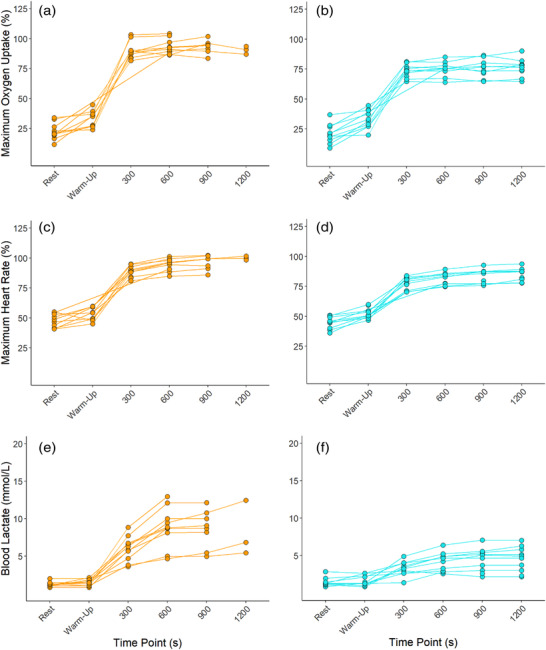
Individual (orange: HVY_TRAD_; blue: HVY_THR_) responses in oxygen uptake expressed relative to maximum oxygen uptake (a, b), heart rate expressed relative to maximum heart rate (c, d), and blood lactate (e, f).

Completion rates for HVY_THR_ and HVY_TRAD_ were 100% and 30%, respectively. In the three individuals who completed HVY_TRAD_, the work rates associated with 75% ${\dot{V}}_{O_{2}\max}$ were below or at CP (96–100% CP). For these individuals, the intensity elicited was primarily consistent with heavy intensity exercise whereby exercise can be continued for extended periods of time with physiological perturbations reaching a delayed steady‐state (Poole et al., 2016). In the seven individuals who were not able to complete HVY_TRAD_, work rates were all above CP (101–132% CP). Exercising above CP elicits non steady‐state exercise and continuation in this domain leads to the eventual attainment of ${\dot{V}}_{O_{2}\max}$ and, ultimately, exhaustion (Poole et al., 2016). Accordingly, in those who were not able to complete HVY_TRAD_ and were exercising >CP, end ${\dot{V}}_{O_{2}}$ and HR values reached ∼95% ${\dot{V}}_{O_{2}\max}$ and ∼97% HR_max_, respectively. In comparison, all individuals were able to complete HVY_THR_ and were all exercising <CP. Accordingly, end ${\dot{V}}_{O_{2}}$ and HR values in HVY_THR_ were ∼76% ${\dot{V}}_{O_{2}\max}$ and ∼85% HR_max_, respectively. This highlights the disparity between the prescribed work rates and the actual work rates elicited through TRAD compared to THR prescription methods. Furthermore, compared to HVY_THR_ where only one individual saw an increase of Bla > 1 mmol l^−1^ from 600 s to 1200 s, four individuals saw an increase >1 mmol l^−1^ from 600 s to 1200 s in HVY_TRAD_ (Figure 3). Exercising at 50% ∆, thus, better normalised exercise intensity among individuals, controlling exercise intensity in the heavy intensity domain. This approach also elicited 46% less variability in work rates (*F = 0.234*). Overall, these findings are consistent with those of Lansley et al. (2011), whereby four individuals (44%) could not complete 20 min of exercise at 70% ${\dot{V}}_{O_{2}\max}$, all reaching ${\dot{V}}_{O_{2}\max}$ and volitional exhaustion before 20 min had elapsed. Similarly, Scharhag‐Rosenberger et al. (2010) noted two (10%) and 17 (81%) individuals were not able to complete 60 min continuous exercise at 60% and 75% ${\dot{V}}_{O_{2}\max}$, respectively. It is thus clear that using a fixed %${\dot{V}}_{O_{2}\max}$ does not control exercise intensity effectively among individuals.

**FIGURE 4:**
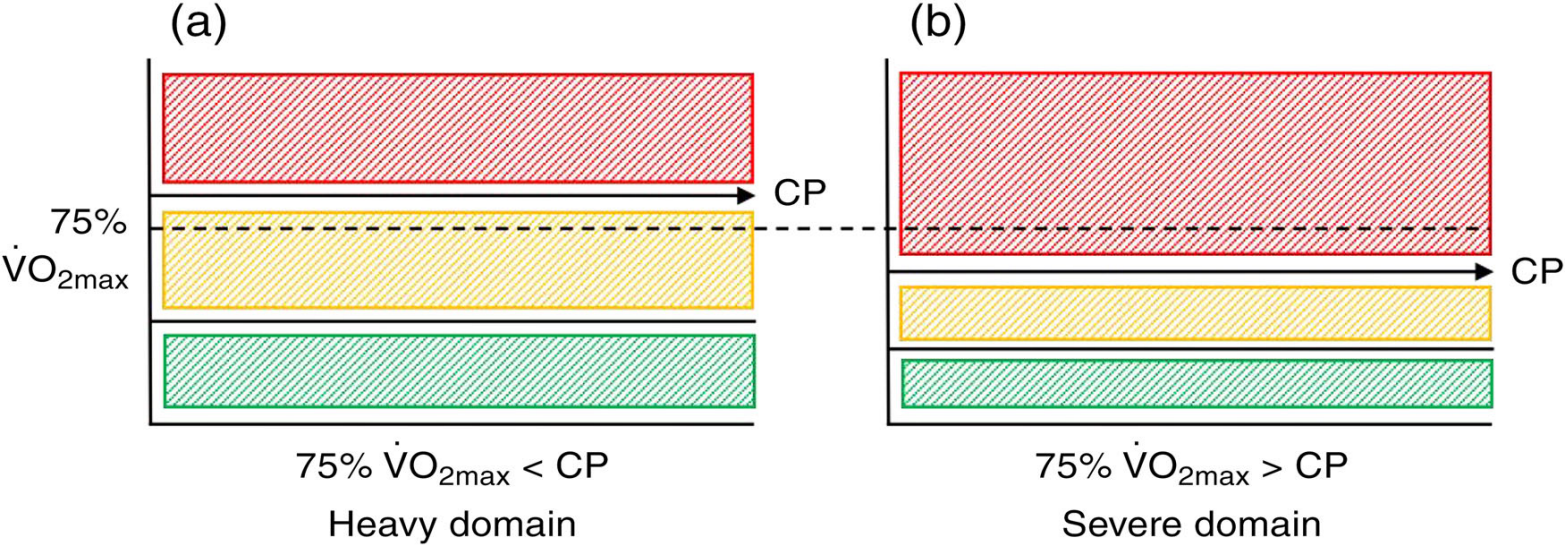
Intensity domain distribution from two representative individuals from the present study. For Individual (a), critical power (CP) occurs at a higher percentage of maximum oxygen uptake (${\dot{V}}_{O_{2}\max}$) compared to person (b). When prescribed exercise at 75% ${\dot{V}}_{O_{2}\max}$, for person (a) this elicited heavy intensity exercise but severe intensity exercise for person (b). If exercise is prescribed relative to CP, this considers the positioning of CP relative to the individual's ${\dot{V}}_{O_{2}\max}$.

Notably, the physiological thresholds which delineate the intensity domains occur at different percentages of ${\dot{V}}_{O_{2}\max}$ among individuals (Azevedo et al., 2011; Hansen et al., 2019; Pymer et al., 2020). Thus, by using physiological thresholds to inform intensity prescription, the size and positioning of an individual's intensity domains are considered (Figure 4). In the present study, when exercising at 75% ${\dot{V}}_{O_{2}\max}$, which is commonly but erroneously assumed to elicit heavy intensity exercise at the individual level, this resulted in exercise undertaken above CP for 70% of individuals, and elicited severe intensity responses to exercise. This corroborates the work of Collins et al. (2022) whereby exercise prescribed at 40% and 80% of GXT maximum power output elicited work rates of 60–72% and 109–148% CP, respectively. Combined with the present findings, this further advocates the use of CP as a primary anchor of exercise intensity. Due to the variability in work rates expressed relative to CP when intensity is prescribed using a fixed %${\dot{V}}_{O_{2}\max}$, future work should determine whether the greater heterogeneity in the exercise stimulus contributes to the commonly observed ${\dot{V}}_{O_{2}\max}$ response variability following a period of traditionally prescribed training.

Unlike Lansley et al. (2011), who observed lower inter‐individual variability in the acute cardiopulmonary responses to exercise at 40% ∆ (where ∆ was determined as GET + [0.4 × (${\dot{V}}_{O_{2}\max}$ − GET)]) compared to 70% ${\dot{V}}_{O_{2}\max}$, no such differences were observed in the present study between HVY_THR_ and HVY_TRAD_ sessions (Figure 3). Based on the marked differences in exercise tolerance in HVY_TRAD_ and HVY_THR_, it is surprising that no additional differences in metabolic or cardiopulmonary response variability were observed.

**FIGURE 5:**
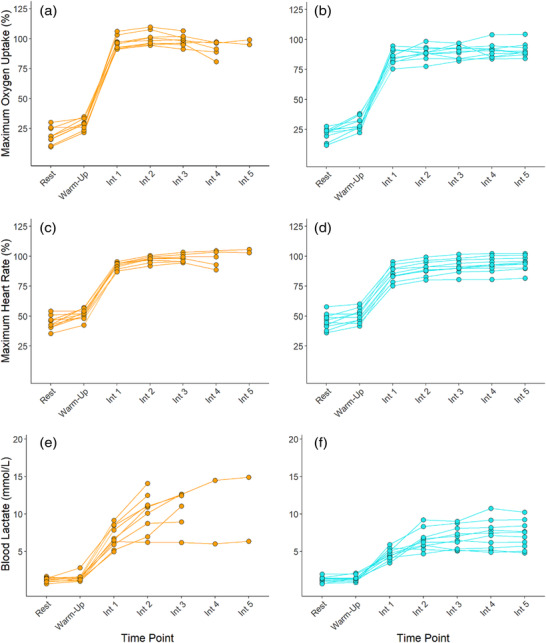
Individual (orange: HIIT_TRAD_; blue: HIIT_THR_) responses in oxygen uptake expressed relative to maximum oxygen uptake (a, b), heart rate expressed relative to maximum heart rate (c, d), and blood lactate (e, f). Int: severe intensity interval bout.

**FIGURE 6:**
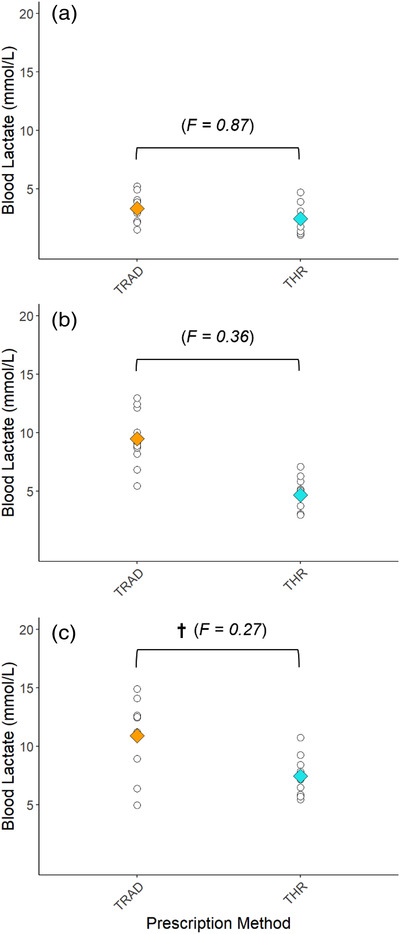
Individual (white circles) and mean (diamonds, orange: TRAD; blue: THR) values for average blood lactate during MOD (a) and peak blood lactate values during HVY (B) and HIIT (c). †Lower variability in THR versus TRAD exercise (*F* < 0.33). *n* = 10.

Completion rates for HIIT_THR_ and HIIT_TRAD_ were 100% and 20%, respectively. In HIIT_TRAD_, two subjects completed all five intervals, four completed four intervals, three completed three intervals, and one individual completed one interval (Figure 5). This demonstrates the large variability in the exercise stimulus elicited when exercising at a work rate corresponding with 85% ${\dot{V}}_{O_{2}\max}$ compared to that of 110% CP. Compared to all individuals exercising at 110% CP in HIIT_THR_, work rates ranged between 115% and 156% CP in HIIT_TRAD_, explaining the variability in time to task failure demonstrated in Figure 5. This is noteworthy given recent findings by Collins et al. (2022) whereby changes in endurance performance were influenced strongly by the intensity of the exercise programme when expressed relative to CP. The variability in peak and average BLa responses to HIIT_THR_ were 53% (*F* = 0.274) and 56% (*F* = 0.318) lower than those in HIIT_TRAD_, respectively (Table 4, Figure 6). Observing no differences in HR and ${\dot{V}}_{O_{2}}$ response variability between HIIT sessions may be explained by a ceiling effect whereby the physiological parameters approach their maximum values and thus room for variance begins to diminish. The observation of reductions in individuals’ ${\dot{V}}_{O_{2}}$ from the last completed bout to that eliciting task failure (Figure 5) is likely explained by the shorter exercise time and thus a shortened amount of time in which ${\dot{V}}_{O_{2}}$ can rise.

**FIGURE 7:**
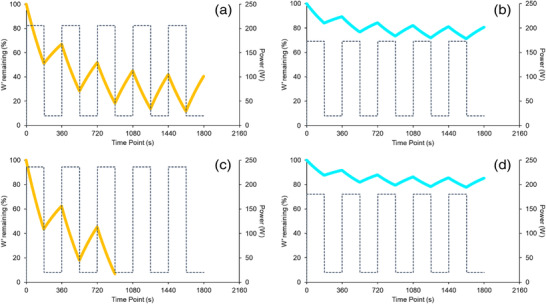
*W′* balance during HIIT_TRAD_ (orange) and HIIT_THR_ (blue) for an individual who completed both HIIT_TRAD_ and HIIT_THR_ (a, b) and for an individual who completed HIIT_THR_ but not HIIT_TRAD_ (c, d).

In the present study, the *W′*_BAL‐INT_ model was used retrospectively (Figure 7). However, this model can be used to design and prescribe HIIT sessions (Galán‐Rioja et al., 2022), for example, designing and prescribing sessions for each individual that target a given *W′* depletion at the end of bout 1 or at the end of the final bout. Despite not doing so in the present study, 5 × 3 min at 110% CP was effective in creating a more homogeneous exercise stimulus than that of HIIT_TRAD_. For example, *W′* depleted at the end of HIIT_THR_ was 30 ± 12% compared to 73 ± 22% in HIIT_TRAD_, a lower variability of 55% (*F* = 0.305). This helps explain the greater variability observed in exercise tolerance following HIIT_TRAD_ and further highlights the disadvantages of using fixed %${\dot{V}}_{O_{2}\max}$ to prescribe exercise. It is of interest to determine whether using the *W′*_BAL‐INT_ model to design and prescribe HIIT_THR_ further amplifies the reduction in response variability to HIIT sessions and enables the prescription of more challenging but achievable interval sessions.

Whilst the addition of CP determination can be time costly and requires the means of determining power output, the marked benefit it has on exercise intensity control is arguably justified. Alternatively, the 3‐min all‐out test has been established as a time‐efficient alternative to the traditional means of determining CP; however, this requires large amounts of motivation, and a familiarisation session is recommended in order to obtain reliable data thereafter (Vanhatalo et al., 2007). Alternatively, determining critical speed, the running analogue of CP, is somewhat easier as this can be determined from training data (i.e., performance or training bests for a given distance) which does not require laboratory equipment beyond a stopwatch and a measure of distance (Smyth & Muniz‐Pumares, 2020). Recent studies are exploring the use of self‐assessed threshold tools such as rate of perceived exertion and the ‘Talk Test’ to estimate individuals’ physiological thresholds (Lehtonen et al., 2022; Preobrazenski et al., 2019). This is an interesting avenue aiming to encourage the rollout of individualised, population‐wide approaches of exercise prescription that do not require access to laboratory facilities (Lehtonen et al., 2022). Additionally, the benefit of using such approaches is also being realised for use in various clinical populations (Anselmi et al., 2021; D'Ascenzi et al., 2022; Mezzani et al., 2013; Pymer et al., 2020).

Finally, whilst it is recommended that practitioners prescribe exercise interventions known to elicit the largest mean changes in ${\dot{V}}_{O_{2}\max}$ in order to maximise the number of individuals experiencing clinically important cardiorespiratory changes (Bonafiglia, 2022), using physiological thresholds to anchor exercise intensity may have a similar effect, without having to exhaust training volume whereby a more appropriate exercise stimulus is created from the beginning.

### 4.1 Conclusions

Overall, prescribing exercise relative to ${\dot{V}}_{O_{2}\max}$ consistently overestimated the boundary between the heavy and severe intensity domains in the present study, in turn causing greater heterogeneity in exercise tolerance and metabolic responses to exercise. More routine testing of individuals’ CP is thus encouraged such that CP can be used to inform and prescribe exercise more appropriately. Future research exploring the feasibility and manipulation of CP determination across different populations is recommended.

### 4.2 Perspective

Due to the widespread usage of traditional intensity anchors (e.g., %${\dot{V}}_{O_{2}\max}$) in training programmes and exercise research studies, it is plausible that this contributes to a heterogeneous training stimulus and thus, at least in part, the variability in physiological outcomes. This may have large implications on longer term training adaptations and the variability of these adaptations among individuals. Future research determining whether this is the case is encouraged. If improving exercise intensity control by use of physiological thresholds does reduce the variability in subsequent exercise‐induced adaptations among individuals, this could have marked benefits on improving exercise interventions and increasing the number of individuals attaining the desired exercise‐induced adaptations targeting both health‐ and performance‐related outcomes.

## AUTHOR CONTRIBUTIONS

Samuel Meyler drafted the manuscript and Daniel Muniz‐Pumares, Lindsay Bottoms, David Wellsted were all involved in the editing of the manuscript. All authors have read and approved the final version of this manuscript and agree to be accountable for all aspects of the work in ensuring that questions related to the accuracy or integrity of any part of the work are appropriately investigated and resolved. All persons designated as authors qualify for authorship, and all those who qualify for authorship are listed.

## CONFLICT OF INTEREST

Samuel Meyler, Lindsay Bottoms, David Wellsted and Daniel Muniz‐Pumares declare that they have no conflicting interests. The results of the present study are presented clearly, honestly, and without fabrication, falsification, or inappropriate data manipulation.

## FUNDING INFORMATION

There was no funding used for the current research study.

## Supporting information

Statistical Summary Document

## Data Availability

Data is available upon reasonable request.

## References

[eph13306-bib-0001] Adams, G. S. , Converse, B. A. , Hales, A. H. , & Klotz, L. E. (2021). People systematically overlook subtractive changes. Nature, 592(7853), 258–261. 33828317 10.1038/s41586-021-03380-y

[eph13306-bib-0002] American College of Sports Medicine . (2017). ACSM's guidelines for exercise testing and prescription. 10th ed. Lippincott Williams & Wilkins. 10.1249/JSR.0b013e31829a68cf 23851406

[eph13306-bib-0003] Anselmi, F. , Cavigli, L. , Pagliaro, A. , Valente, S. , Valentini, F. , Cameli, M. , Focardi, M. , Mochi, N. , Dendale, P. , Hansen, D. , Bonifazi, M. , Halle, M. , & D'Ascenzi, F. (2021). The importance of ventilatory thresholds to define aerobic exercise intensity in cardiac patients and healthy subjects. Scandinavian Journal of Medicine & Science in Sports, 31(9), 1796–1808. 34170582 10.1111/sms.14007 PMC8456830

[eph13306-bib-0004] Azevedo, L. F. , Perlingeiro, P. S. , Brum, P. C. , Braga, A. M. W. , Negrão, C. E. , & de Matos, L. D. N. J. (2011). Exercise intensity optimization for men with high cardiorespiratory fitness. Journal of Sports Sciences, 29(6), 555–561. 21360401 10.1080/02640414.2010.549613

[eph13306-bib-0005] Baldwin, J. , Snow, R. J. , & Febbraio, M. A. (2000). Effect of training status and relative exercise intensity on physiological responses in men. Medicine and Science in Sports and Exercise, 32(9), 1648–1654. 10994919 10.1097/00005768-200009000-00020

[eph13306-bib-0006] Bassett, D. R. , & Howley, E. T. (2000). Limiting factors for maximum oxygen uptake and determinants of endurance performance. Medicine and Science in Sports and Exercise, 32(1), 70–84. 10647532 10.1097/00005768-200001000-00012

[eph13306-bib-0007] Black, M. I. , Jones, A. M. , Blackwell, J. R. , Bailey, S. J. , Wylie, L. J. , McDonagh, S. T. J. , Thompson, C. , Kelly, J. , Sumners, P. , Mileva, K. N. , Bowtell, J. L. , & Vanhatalo, A. (2017). Muscle metabolic and neuromuscular determinants of fatigue during cycling in different exercise intensity domains. Journal of Applied Physiology, 122(3), 446–459. 28008101 10.1152/japplphysiol.00942.2016 PMC5429469

[eph13306-bib-0008] Bonafiglia, J. T. , Preobrazenski, N. , Islam, H. , Walsh, J. J. , Ross, R. , Johannsen, N. M. , Martin, C. K. , Church, T. S. , Slentz, C. A. , Ross, L. M. , Kraus, W. E. , Kenny, G. P. , Goldfield, G. S. , Prud'homme, D. , Sigal, R. J. , Earnest, C. P. , & Gurd, B. J. (2021). Exploring differences in cardiorespiratory fitness response rates across varying doses of exercise training: A retrospective analysis of eight randomized controlled trials. Sports Medicine, 51(8), 1785–1797. 33704698 10.1007/s40279-021-01442-9

[eph13306-bib-0009] Bonafiglia, J. T. , Swinton, P. A. , Ross, R. , Johannsen, N. M. , Martin, C. K. , Church, T. S. , Slentz, C. A. , Ross, L. M. , Kraus, W. E. , Walsh, J. J. , Kenny, G. P. , Goldfield, G. S. , Prud'homme, D. , Sigal, R. J. , Earnest, C. P. , & Gurd, B. J. (2022). Interindividual differences in trainability and moderators of cardiorespiratory fitness, waist circumference, and body mass responses: A large‐scale individual participant data Meta‐analysis. Sports Medicine, 52(12), 2837–2851. 35781787 10.1007/s40279-022-01725-9

[eph13306-bib-0010] Bouchard, C. , An, P. , Rice, T. , Skinner, J. S. , Wilmore, J. H. , Gagnon, J. , Pérusse, L. , Leon, A. S. , & Rao, D. C. (1999). Familial aggregation of ${\dot{V}}_{O_{2}\max}$ response to exercise training: Results from the Heritage family study. Journal of Applied Physiology, 87(3), 1003–1008. 10484570 10.1152/jappl.1999.87.3.1003

[eph13306-bib-0011] Carter, H. , Pringle, J. S. M. , Jones, A. M. , & Doust, J. H. (2002). Oxygen uptake kinetics during treadmill running across exercise intensity domains. European Journal of Applied Physiology, 86(4), 347–354. 11990749 10.1007/s00421-001-0556-2

[eph13306-bib-0012] Chen, S. , & Chen, H. (2010). Cohen's f statistics. In N. Salkind (Ed.), Encyclopedia of research design. SAGE Publications, Inc.

[eph13306-bib-0013] Collins, J. , Leach, O. , Dorff, A. , Linde, J. , Kofoed, J. , Sherman, M. , Proffit, M. , & Gifford, J. R. (2022). Critical power and work‐prime account for variability in endurance training adaptations not captured by ${\dot{V}}_{O_{2}\max}$ . Journal of Applied Physiology, 133(4), 986–1000. 36107986 10.1152/japplphysiol.00344.2022

[eph13306-bib-0014] D'Ascenzi, F. , Cavigli, L. , Pagliaro, A. , Focardi, M. , Valente, S. , Cameli, M. , Mandoli, G. E. , Mueller, S. , Dendale, P. , Piepoli, M. , Wilhelm, M. , Halle, M. , Bonifazi, M. , & Hansen, D. (2022). Clinician approach to cardiopulmonary exercise testing for exercise prescription in patients at risk of and with cardiovascular disease. British Journal of Sports Medicine, 56(20), 1180–1187. 10.1136/bjsports-2021-105261 35680397

[eph13306-bib-0015] Galán‐Rioja, M. Á. , González‐mohíno, F. , Skiba, P. F. , González‐ravé, J. M. , Galán‐rioja, M. Á. , & González‐mohíno, F. (2022). Utility of the W´_BAL_ model in training programme design for masters cyclists. European Journal of Sport Science, 1–10. 36310098 10.1080/17461391.2022.2142675

[eph13306-bib-0016] Hansen, D. , Bonné, K. , Alders, T. , Hermans, A. , Copermans, K. , Swinnen, H. , Maris, V. , Jansegers, T. , Mathijs, W. , Haenen, L. , Vaes, J. , Govaerts, E. , Reenaers, V. , Frederix, I. , & Dendale, P. (2019). Exercise training intensity determination in cardiovascular rehabilitation: Should the guidelines be reconsidered? European Journal of Preventive Cardiology, 26(18), 1921–1928. 31219704 10.1177/2047487319859450

[eph13306-bib-0017] Harber, M. P. , Kaminsky, L. A. , Arena, R. , Blair, S. N. , Franklin, B. A. , Myers, J. , & Ross, R. (2017). Impact of cardiorespiratory fitness on all‐cause and disease‐specific mortality: Advances since 2009. Progress in Cardiovascular Diseases, 60(1), 11–20. 28286137 10.1016/j.pcad.2017.03.001

[eph13306-bib-0018] Hunter, B. , Greenhalgh, A. , Karsten, B. , Burnley, M. , & Muniz‐Pumares, D. (2021). A non‐linear analysis of running in the heavy and severe intensity domains. European Journal of Applied Physiology, 121(5), 1297–1313. 33580289 10.1007/s00421-021-04615-6

[eph13306-bib-0019] Iannetta, D. , Inglis, E. C. , Mattu, A. T. , Fontana, F. Y. , Pogliaghi, S. , Keir, D. A. , & Murias, J. M. (2020). A critical evaluation of current methods for exercise prescription in women and men. Medicine and Science in Sports and Exercise, 52(2), 466–473. 31479001 10.1249/MSS.0000000000002147

[eph13306-bib-0020] Jones, A. M. , Burnley, M. , Black, M. I. , Poole, D. C. , & Vanhatalo, A. (2019). The maximal metabolic steady state: Redefining the ‘gold standard’. Physiological Reports, 7(10), 1–16. 10.14814/phy2.14098 PMC6533178 31124324

[eph13306-bib-0021] Keir, D. A. , Iannetta, D. , Mattioni Maturana, F. , Kowalchuk, J. M. , & Murias, J. M. (2022). Identification of non‐invasive exercise thresholds: Methods, strategies, and an online app. Sports Medicine, 52(2), 237–255. 34694596 10.1007/s40279-021-01581-z

[eph13306-bib-0022] Lansley, K. E. , Dimenna, F. J. , Bailey, S. J. , & Jones, A. M. (2011). A new method to normalise exercise intensity. International Journal of Sports Medicine, 32(07), 535–541. 21563028 10.1055/s-0031-1273754

[eph13306-bib-0023] Lehtonen, E. , Gagnon, D. , Eklund, D. , Kaseva, K. , & Peltonen, J. E. (2022). Hierarchical framework to improve individualised exercise prescription in adults: A critical review. BMJ Open Sport & Exercise Medicine, 8(2), e001339. 10.1136/bmjsem-2022-001339 PMC9185660 35722045

[eph13306-bib-0024] McLellan, T. M. , & Jacobs, I. (1991). Muscle glycogen utilization and the expression of relative exercise intensity. International Journal of Sports Medicine, 12(1), 21–26. 2030054 10.1055/s-2007-1024649

[eph13306-bib-0025] Meyer, T. , Gabriel, H. H. W. , & Kindermann, W. (1999). Is determination of exercise intensities as percentages of ${\dot{V}}_{O_{2}\max}$ or HRmax adequate? Medicine and Science in Sports and Exercise, 31(9), 1342–1345. 10487378 10.1097/00005768-199909000-00017

[eph13306-bib-0026] Meyler, S. , Bottoms, L. , & Muniz‐Pumares, D. (2021). Biological and methodological factors affecting ${\dot{V}}_{O_{2}\max}$ response variability to endurance training and the influence of exercise intensity prescription. In Experimental physiology (p. 1410). John Wiley & Sons, Ltd. 10.1113/EP089565 34036650

[eph13306-bib-0027] Mezzani, A. , Hamm, L. F. , Jones, A. M. , McBride, P. E. , Moholdt, T. , Stone, J. A. , Urhausen, A. , & Williams, M. A. (2013). Aerobic exercise intensity assessment and prescription in cardiac rehabilitation: A joint position statement of the European association for cardiovascular prevention and rehabilitation, the American association of cardiovascular and pulmonary rehabilitation and the Canadian association of cardiac rehabilitation. European Journal of Preventive Cardiology, 20(3), 442–467. 23104970 10.1177/2047487312460484

[eph13306-bib-0028] Milanović, Z. , Sporiš, G. , & Weston, M. (2015). Effectiveness of high‐intensity interval training (HIT) and continuous endurance training for VO2max improvements: A systematic review and meta‐analysis of controlled trials. Sports Medicine, 45(10), 1469–1481. 26243014 10.1007/s40279-015-0365-0

[eph13306-bib-0029] Muniz‐Pumares, D. , Karsten, B. , Triska, C. , & Glaister, M. (2019). Methodological approaches and related challenges associated with the determination of critical power and curvature constant. Journal of Strength and Conditioning Research, 33(2), 584–596. 30531413 10.1519/JSC.0000000000002977

[eph13306-bib-0030] Nolan, P. B. , Beaven, M. L. , & Dalleck, L. (2014). Comparison of intensities and rest periods for VO2max verification testing procedures. International Journal of Sports Medicine, 35(12), 1024–1029. 24886925 10.1055/s-0034-1367065

[eph13306-bib-0031] Poole, D. C. , Burnley, M. , Vanhatalo, A. , Rossiter, H. B. , & Jones, A. M. (2016). Critical power: An important fatigue threshold in exercise physiology. Medicine and Science in Sports and Exercise, 48(11), 2320–2334. 27031742 10.1249/MSS.0000000000000939 PMC5070974

[eph13306-bib-0032] Poole, D. C. , & Jones, A. M. (2017). Measurement of the maximum oxygen uptake Vo2max: Vo2peak is no longer acceptable. Journal of Applied Physiology, 122(4), 997–1002. 28153947 10.1152/japplphysiol.01063.2016

[eph13306-bib-0033] Poole, D. C. , Rossiter, H. B. , Brooks, G. A. , & Gladden, L. B. (2020). The anaerobic threshold: 50+ years of controversy. Journal of Physiology, 599(3), 737–767. 33112439 10.1113/JP279963

[eph13306-bib-0043] Preobrazenski, N. , Bonafiglia, J. T. , Nelms, M. W. , Lu, S. , Robins, L. , LeBlanc, C. , & Gurd, B. J. (2019). Does blood lactate predict the chronic adaptive response to training: A comparison of traditional and talk test prescription methods. Applied Physiology, Nutrition, and Metabolism, 44(2), 179–186. 10.1139/apnm-2018-0343 30058347

[eph13306-bib-0034] Pymer, S. , Nichols, S. , Prosser, J. , Birkett, S. , Carroll, S. , & Ingle, L. (2020). Does exercise prescription based on estimated heart rate training zones exceed the ventilatory anaerobic threshold in patients with coronary heart disease undergoing usual‐care cardiovascular rehabilitation? A United Kingdom perspective. European Journal of Preventive Cardiology, 27(6), 579–589. 31116574 10.1177/2047487319852711

[eph13306-bib-0035] Scharhag‐Rosenberger, F. , Meyer, T. , Gäßler, N. , Faude, O. , & Kindermann, W. (2010). Exercise at given percentages of VO2max: Heterogeneous metabolic responses between individuals. Journal of Science and Medicine in Sport, 13(1), 74–79. 19230766 10.1016/j.jsams.2008.12.626

[eph13306-bib-0036] Skiba, P. F. , & Clarke, D. C. (2021). The W′ balance model: Mathematical and methodological considerations. International Journal of Sports Physiology and Performance, 16(11), 1561–1572. 34686611 10.1123/ijspp.2021-0205

[eph13306-bib-0037] Smyth, B. , & Muniz‐Pumares, D. (2020). Calculation of critical speed from raw training data in recreational marathon runners. Medicine and Science in Sports and Exercise, 52(12), 2637–2645. 32472926 10.1249/MSS.0000000000002412 PMC7664951

[eph13306-bib-0038] Vanhatalo, A. , Doust, J. H. , & Burnley, M. (2007). Determination of critical power using a 3‐min all‐out cycling test. Medicine and Science in Sports and Exercise, 39(3), 548–555. 17473782 10.1249/mss.0b013e31802dd3e6

[eph13306-bib-0039] Wasserman, K. , Whipp, B. J. , Koyal, S. N. , & Beaver, W. L. (1973). Anaerobic threshold and respiratory gas exchange during exercise. Journal of Applied Physiology, 35(2), 236–243. 4723033 10.1152/jappl.1973.35.2.236

[eph13306-bib-0040] Wen, D. , Utesch, T. , Wu, J. , Robertson, S. , Liu, J. , Hu, G. , & Chen, H. (2019). Effects of different protocols of high intensity interval training for VO2max improvements in adults: A meta‐analysis of randomised controlled trials. Journal of Science and Medicine in Sport, 22(8), 941–947. 30733142 10.1016/j.jsams.2019.01.013

[eph13306-bib-0041] Williams, C. J. , Gurd, B. J. , Bonafiglia, J. T. , Voisin, S. , Li, Z. , Harvey, N. , Croci, I. , Taylor, J. L. , Gajanand, T. , Ramos, J. S. , Fassett, R. G. , Little, J. P. , Francois, M. E. , Hearon, C. M. , Sarma, S. , Janssen, S. L. J. E. , van Craenenbroeck, E. M. , Beckers, P. , Cornelissen, V. A. , … Coombes, J. S. (2019). A multi‐center comparison of VO2peak trainability between interval training and moderate intensity continuous training. Frontiers in Physiology, 10, 19. 30804794 10.3389/fphys.2019.00019 PMC6370746

