# Supplementary material for: Variability in exercise tolerance and physiological responses to exercise prescribed relative to physiological thresholds and to maximum oxygen uptake
Source: Exp Physiol. 2023 Jan 29;108(4):581–94. doi: 10.1113/EP090878 (PMC10103872; doi:10.1113/EP090878)
Supplement: Supplementary file 1 — Statistical Summary Document [file EPH-108-581-s001.docx]

**Manuscript Title:** Variability in exercise tolerance and physiological responses to exercise prescribed relative to physiological thresholds and to maximum oxygen uptake

**Authors:** Samuel Meyler, Lindsay Bottoms, David Wellsted, Daniel Muniz-Pumars

**Animal model used, if applicable:**

**Underlying hypothesis:** The study investigated whether the variability in exercise tolerance and acute physiological responses were lower when exercise bouts are prescribed relative to physiological thresholds compared to when prescribed relative to maximum oxygen uptake

**Definitions of ‘n’:**

N = number of individuals

**Statistical summary table:**

| Experimental question number* | Finding/ conclusion | Experimental location/ variable  e.g. muscle, neocortex or genotype | Mean value  (or other summary statistic) | SD | n val. | P** | Units | Data comparisons  e.g. WT vs KO | Statistical test | Any other variable  e.g. subjects’ age or sex | Figure/ table in which data are presented | Comments  e.g. observation |
| --- | --- | --- | --- | --- | --- | --- | --- | --- | --- | --- | --- | --- |
| 1 | Lower completion rates in HVY TRAD vs HVY THR | Completion rate | 30% (TRAD) 100% (THR) | nA | 10 | P <0.001 | % | THR v TRAD | Chi-sq test |  | Table 3 |  |
| 2 | Lower completion rates in HIIT TRAD vs HIIT THR | Completion rate | 20% (TRAD) 100% (THR) | nA | 10 | P <0.001 | % | THR v TRAD | Chi-sq test |  | Table 3 |  |
| 3 | Lower work rate variance in HVY THR vs HVY TRAD | % CP | 83 (THR) 113 (TRAD) | 6 (THR) 11 (TRAD) | 10 | F = 0.234 (F stat as ES) | % | THR v TRAD | F test |  | Table 3 |  |
| 4 | Lower work rate variance in HIIT THR vs HIIT TRAD | % CP | 110 (THR) 134 (TRAD) | 0 (THR) 15 (TRAD) | 10 | F <0.001 (F stat as ES) | % | THR v TRAD | F test |  | Table 3 |  |
| 5 | Lower variability in peak blood lactate responses in HIIT THR vs HIIT TRAD | Blood lactate | 7.45 (THR) 10.91 (TRAD) | 1.70 (THR) 3.23 (TRAD) | 10 | F = 0.274 (F stat as ES) | mmol/L | THR v TRAD | F test |  | Table 4 |  |
| 6 | Lower variability in average blood lactate responses in HIIT THR vs HIIT TRAD | Blood lactate | 6.50 (THR) 9.09 (TRAD) | 1.30 (THR) 2.31 (TRAD) | 10 | F = 0.318 (F stat as ES) | mmol/L | THR v TRAD | F test |  | Table 4 |  |
| 7 | W' depletion at the end of interval 1 was greater in HIIT TRAD vs HIIT THR | W' depletion | 17 (THR) 49 (TRAD) | 7 (THR) 7 (TRAD) | 10 | P <0.001 | KJ | THR v TRAD | T test |  | Results |  |
| 8 | W' depletion at the end of the final interval was greater in HIIT TRAD vs HIIT THR | W' depletion | 30 (THR) 73 (TRAD) | 12 (THR) 22 (TRAD) | 10 | P <0.001 | KJ | THR v TRAD | T test |  | Results |  |
| 9 | Lower variability in W'bal available at the end of HIIT in HIIT THR vs HIIT TRAD | W' depletion | 30 (THR) 73 (TRAD) | 12 (THR) 22 (TRAD) | 10 | F = 0.305 (F stat as ES) | KJ | THR v TRAD | T test |  | Results |  |
|  |  |  |  |  |  |  |  |  |  |  |  |  |

*You may use multiple lines for the same question to indicate multiple comparisons

** Authors may wish to make the text bold where p is considered significant against a stated confidence limit.
